# Time perspective predicts levels of anxiety and depression during the COVID-19 outbreak: A cross-cultural study

**DOI:** 10.1371/journal.pone.0269396

**Published:** 2022-09-29

**Authors:** Luigi Micillo, Pier-Alexandre Rioux, Esteban Mendoza, Sebastian L. Kübel, Nicola Cellini, Virginie Van Wassenhove, Simon Grondin, Giovanna Mioni

**Affiliations:** 1 Department of General Psychology, University of Padova, Padova, Italy; 2 École de Psychologie, Université Laval, Québec, QC, Canada; 3 Institute for Frontier Areas of Psychology and Mental Health, Freiburg, Germany; 4 Max Planck Institute for the Study of Crime, Security and Law, Freiburg, Germany; 5 Padova Neuroscience Center, University of Padova, Padova, Italy; 6 Human Inspired Technology Center, University of Padova, Padova, Italy; 7 Cognitive Neuroimaging Unit, NeuroSpin, CEA, INSERM, CNRS, Université Paris-Saclay, Gif/Yvette, France; State University of New York Upstate Medical University, UNITED STATES

## Abstract

The COVID-19 outbreak and governmental measures to keep the population safe had a great impact on many aspects of society, including well-being. Using data from N = 1281 participants from six countries (Argentina, France, Greece, Italy, Japan, and Turkey), we first explored differences in anxiety, depression (measured with the Hospital Anxiety and Depression Scale; HADS), and time perspectives (Zimbardo Time Perspective Inventory; ZTPI), between these countries during the first weeks of the pandemic. We observed that Turkish participants reported the highest levels of anxiety, and Japanese and Greek the lowest. For depression symptoms, the Japanese scored highest and Italians lowest. Next, for each country, we investigated how well the relatively time-stable personality traits of time perspectives, chronotype (reduced Morningness-Eveningness Questionnaire; rMEQ), and Big Five personality traits (short Big Five Inventory; BFI) predicted the levels of anxiety and depression (HADS). The regression analyses showed that negative attitudes towards the past predicted the levels of both anxiety and depression in most of the countries we analyzed. Additionally, in many countries, a Past Positive orientation negatively predicted depression whereas the Present Fatalistic subscale predicted anxiety and depression. The chronotype did not contribute additionally to the models. The Big Five traits (and particularly neuroticism) showed substantial incremental explanatory power for anxiety in some countries but did not consistently predict anxiety levels. For depression, the additional variance accounted for by including the BFI as predictors was rather small. Importantly, the ZTPI subscales were retained as significant predictors in the model still when the BFI and rMEQ were considered as potential predictors. Our results yield evidence that the ZTPI time perspectives are valuable predictors for anxiety and depression levels during the first period of the pandemic.

## Introduction

### Psychological consequences of the pandemic situation

Facing the Covid-19 pandemic, governments around the world felt compelled to restrict citizens’ mobility to protect the population. Besides its effectiveness for the containment of the virus, social distancing has consequences for both short- and long-term well-being [1]. The pandemic situation can affect various psychological outcomes: people reported experiencing more fear of getting sick, being hopeless, or being stereotyped by others [2]. A review by Brooks and colleagues [2] concluded that being quarantined was associated with negative emotions such as anxiety and depression. In a systematic review, Salari and colleagues [3] analyzed 17 studies from nine countries conducted during the COVID-19 outbreak and showed that the pandemic affected mental health and raised the levels of depression, anxiety, and stress. This increase can be explained by multiple factors: for instance, the World Health Organization [4] asserted that the more people were exposed to (potentially distressful) news on COVID-19, the higher the reported levels of anxiety and depression were, unless the information were accurate and up-to-date [5]. The highest levels of anxiety and depression were found in women, young adults, individuals with higher levels of education, and Asians as compared to Europeans [3].

The pandemic and the associated routines also affected the perception of time: agendas and routines changed because of the loss of zeitgebers and temporal landmarks, up to a level where the days lost their identity and significance [4, 6]. In the UK, Ogden [7] observed that approximately 40% of the participants indicated that time passed more slowly, and 40% felt it passed more quickly than before the lockdown. Individuals who were more satisfied with their social situation and engaged in more activities perceived the passage of time as more quickly compared to before the lockdown. Similarly, in France, boredom and sadness predicted a perceived slowing down of the passage of time [8]. Besides forgetting what day it was, the lockdown led to paradoxical impressions that time flew slowly, but in hindsight intervals were perceived as short, or the other way around [9]. For some, monotony during the quarantine induced boredom, leading to a perceived deceleration of the passage of time. However, with few significant memories, intervals appear to be shorter in hindsight [10]. For others, attention might be attracted by less monotony in the daily structure, by the challenge of organizing (more) activities (e.g., due to additional childcare duties), and by the occurring events during the first phase of the quarantine. With less attention directed to time, time is perceived as passing more quickly [4]. However, when intervals are then judged retrospectively, they are estimated longer when they are filled with less routine activities and when more unique memories can be retrieved [10].

Next to this altered perception of how fast time flies, the pandemic situation created uncertainty about how long the lockdown might persist and uncertainty about one’s future prospects. Nobody could predict the duration of the governmental measures, how the pandemic would further affect the health care systems, and what consequences it might have on the global, national, and individual levels concerning the economy and the society, and on one’s own health status. How long will I be in this situation and what are my prospects for the future? Confronted with this (temporal) uncertainty, individual differences in time perspectives may therefore be an important factor accounting for how individuals manage to adapt to the adverse pandemic situation.

### Brief introduction to time perspectives

Time perspective is a personality trait that is relatively stable over time, shaped through learning experiences and cultural influences [11, 12]. The individual characteristics of time perspectives determine how information is considered and evaluated, and affect individual preferences, decision-making, and behavior (e.g., risk-taking, [13]; aggression, [14]). The concept of time perspectives was initially introduced with Zimbardo and Boyd`s [12] Time Perspective Theory. Accordingly, individual time perspective can be defined as *“the often nonconscious process whereby the continual flows of personal and social experiences are assigned to temporal categories*, *or time frames*, *that help to give order*, *coherence*, *and meaning to those events”* [12, p. 1271). The individual time perspective profile can be measured with the Zimbardo Time Perspective Inventory (ZTPI; [12]) which is composed of 56 items divided into five subscales: Past Negative (PN), Past Positive (PP), Present Fatalistic (PF), Present Hedonistic (PH) and Future (F). As claimed by Zimbardo and Boyd [12, 15], each of these temporal frames reflects specific attitudes in individuals. Past Positive refers to a positive and nostalgic image of the past; the Past Negative factor represents a regretful view on past experiences. High levels on the Present Fatalistic factor are common for individuals who are characterized by a hopeless deterministic attitude towards the present limited in controllability whereas Present Hedonistic describes individuals who are more oriented to the enjoyment of the current moment—they are sensation seekers and worry little about future consequences of their behaviors. Highly future-oriented individuals are characterized by goal-oriented behaviors and high consideration of the consequences of their decisions at the expense of immediate gratifications [12, 16, 17]. These five factors can be influenced by culture: Sircova and colleagues [17] reported that the distribution of the time profiles varies across countries. For instance, in France, there was a strong hedonistic orientation towards the present whereas in Turkey authors observed combinations of future-oriented and past negative and present fatalistic types.

### Personality traits as predictors for anxiety and depression

The five time perspective factors are associated with indicators of well-being. In a sample of Belgian older adults, higher levels of Future and Present Hedonistic factors were associated with positive affect [18]. Participants who were more positively oriented towards the past reported a higher level of life satisfaction. In contrast, the Present Fatalistic and Past Negative factors were associated with symptoms of depression [18]. These results were in line with the initial validation study of the Zimbardo Time Perspective Inventory sampling among students [12] and in which the authors found that high scores on the Past Negative and Present Fatalistic factors were moderately associated with both anxiety and depression whereas a Present Hedonistic perspective was weakly but positively correlated with depression. Additionally, Future and Past Positive orientations were found to be weakly negatively correlated with both depression and anxiety. The negative association of Future and anxiety was also supported by a preliminary study conducted in Greece by Papastematelou and colleagues [19] on a sample of male subjects.

It has been found that the chronotype, the individual difference in circadian preference, also predicts the levels of anxiety and depression [20] Specifically, evening orientation is associated with depression [21, 22], whereas results are inconclusive for anxiety. A review analyzing the relationship between chronotype and mental health found that in adults, eveningness was related to higher levels of anxiety [21]. This relationship, however, is not consistently found in adolescent samples [21].

The levels of anxiety and depression can also be predicted by the established Big Five personality dimensions (agreeableness, conscientiousness, extraversion, neuroticism, and openness). The Big Five traits are related to anxiety and depression: In a meta-analysis of 2010, Kotov and colleagues [23] concluded that neuroticism was positively associated with anxiety and depression whereas extraversion and conscientiousness showed a negative correlation with these pathologies.

### The present study

The present study is part of a wider project which involved several laboratories working on time perception and temporal processing from 15 countries. The project aimed at investigating the individuals’ experience of time during the exceptional circumstances of the COVID-19 pandemic longitudinally and cross-culturally and was composed of multiple sessions (for the registry on the Open Science Framework (OSF), see: [24]). We used data from the first session which was conducted during the first wave of the pandemic in spring 2020.

The purpose of the study at hand was, first, to assess time perspectives, levels of depression, and anxiety interculturally during the first weeks of the pandemic and to detect differences between countries. Second, we wanted to investigate whether and to what extent the relatively stable personality traits of Time Perspectives, Big Five Traits, and chronotype could predict the levels of anxiety and depression. Third, we wanted to extract the set of personality traits that best predicted anxiety and depression. We present parsimonious models that best predicted anxiety and depression levels during the pandemic for each country.

By doing so, we are able to present valuable information about the differences between countries in levels of anxiety and depression during the pandemic. Additionally, we can show how well, and which, personality traits can predict these pathologies—giving clues to which risk factors of personality particular attention needs to be paid. Since our data were collected during the first wave of the COVID-19 pandemic, this allows comparing to what extent our results are consistent with the ones reported from research before the 2020 pandemic.

## Methods

### Project

An international online observation study was conducted by members of the Timing Research Forum (TRF) on the GORILLA™ platform. It was designed for examining various aspects of time perception and timing during the first months of the pandemic and the associated governmental measures. Each of the fifteen countries translated the questionnaires and tasks into their native languages and was responsible for the data collection in their country. No minors were allowed to take part in the study. All participants signed a written Informed Consent and–except for Japan–there was no monetary compensation for participating in the study. The Principal Investigator of the multicenter study–Dr. Virginie van Wassenhove–received the approval for the international study from the Ethical Committee of the University of Paris-Saclay (CER-Paris-Saclay-2020-020). The study protocol was also approved by local Ethical Committees in each country and was developed in accordance with the Declaration of Helsinki (2018). For the present study, we employed data from six countries which provided at least 100 participants who completed all questionnaires: Argentina (AR), France (FR), Greece (GR), Italy (IT), Japan (JP), and Turkey (TR).

### Participants

Participants were invited to participate on the website of the Timing Research Forum and via social media pages. Individuals who were declared to have a diagnosed neurological or a psychological disorder were automatically excluded from the data collection. To have more comparable samples between countries, we restricted the sample to participants aged between 18 to 60 years. As can be seen in Table 1, this resulted in a total of N = 1,281 individuals for the analyses, comprising 899 females and 382 males whose age ranged from 18 to 60 years (M = 34.1; SD = 12.3). However, the distributions of age, sex, and the number of participants were unequal across countries (see Table 1). The six countries included in our sample experienced different levels of restrictions during the period in which data were collected as can be seen in Table 2 [for the stringency index see 25].

**Table 1:** Demographic information on the sample, subdivided by country.

Country	N	Sex		Age			Data Collection	
		Female	Male	M	SD	Range	Start	End
Argentina	206	142	64	33.9	10.2	18–60	12/05	08/06
France	474	340	134	40.9	11.8	19–60	10/04	10/05
Greece	173	130	43	30.9	10.2	18–56	18/04	07/05
Italy	170	108	62	26.7	9.93	18–60	20/04	19/05
Japan	87	47	40	40.3	11.4	21–60	01/05	24/05
Turkey	171	132	39	22.8	4.10	18–55	21/04	07/06
ALL	1,281	899	382	34.1	12.3	18–60	10/04	08/06

**Table 2:** Mobility changes and stringency indices during the assessment period by countries and areas.

country	Period start/end	Retail & Recreation M (SD)	Grocery & Pharmacy M (SD)	Residential M (SD)	Transit Stations M (SD)	Parks M (SD)	Work Places M (SD)	Stringency M (SD) [0–100]
AR	12/05 08/06	-70.49% (2.48)	-24.09% (2.80)	19.32% (0.92)	-54.83% (1.70)	-87.45% (1.35)	-31.22% (3.76)	90.32 (0.79)
FR	10/0410/05	-80.45% (3.10)	-39.76% (3.55)	27.52% (1.40)	-77.58% (2.61)	-62.71% (3.03)	-62.53% (3.64)	87.96 (0.00)
GR	18/0407/05	-69.99% (-5.93)	-10.16% (10.24)	20.28% (2.57)	-62.84% (6.17)	-15.89% (17.69)	-52.14% (6.71)	82.32 (4.39)
IT	20/0419/05	-72.02% (9.81)	-41.19% (8.87)	23.39% (4.76)	-67.92% (9.42)	-56.61% (20.05)	-51.82% (9.78)	77.53 (15.24)
JP	01/0524/05	-32.95% (1.81)	-3.10% (0.82)	15.69% (3.47)	-50.17% (5.58)	-5.48% (5.83)	-31.71% (10.98)	44.25 (3.30)
TR	21/0407/06	-60.72% (10.10)	-20.34% (12.10)	19.71% (4.69)	-60.20% (10.71)	-34.53% (17.10)	-48.26% (10.93)	74.17 (4.29)

Note. Changes in mobility are indicated as the percentage of decrease or increase, compared to the baseline, which is the median value from a 5-week period, Jan 3 –Feb 6, 2020, when none of these countries (AR = Argentina; FR = France; GR = Greece; IT = Italy; JP = Japan and TR = Turkey) was affected by the pandemic. Percentages were averaged over the days of assessment. The Stringency Index reflects governmental measures in response to the pandemic and is a composite measure based on nine response indicators including school closures, workplace closures, and travel bans, rescaled to a value from 0 to 100 (100 = strictest). If policies vary at the subnational level, the index is shown as the response level of the strictest sub-region. *Data derived from OurWorldinData*: *https*:*//ourworldindata*.*org/grapher/changes-visitors-covid*?*[for* *the mobility indices (**https*:*//www*.*google*.*com/covid19/mobility/**)]* *https*:*//ourworldindata*.*org/grapher/covid-stringency-index* *[for the stringency index 25]*.

### Measures

Participants provided demographic data on age and sex. Additionally, we used the following four questionnaires.

#### Hospital Anxiety and Depression Scale (HADS)

The Hospital Anxiety and Depression Scale (HADS, 26) consists of 14 items rated on Likert scales ranging from 0 to 3. The questionnaire can be divided into two seven-item subscales (with sum scores ranging from 0 to 21), one measuring the level of anxiety and one gauging depression symptoms. A score higher than 11 indicates elevated levels of anxiety and depression [26], which was also recommended as a cut-off in a later normative data study with a non-clinical sample [27]. Anxiety and Depression scales are moderately correlated (r = .53); reliabilities as measured with Cronbach`s α are good with α = .82 (Anxiety) and α = .77 (Depression; [27]).

#### Zimbardo Time Perspective Inventory (ZTPI)

The Zimbardo Time Perspective Inventory (ZTPI, [12]) is composed of 56 items. Participants evaluate on a Likert scale ranging from 1 = very untrue to 5 = very true to what extent each statement applies to them. The questionnaire contains five subscales derived from factor analytic studies: Past Negative (PN), Past Positive (PP), Present Fatalistic (PF), Present Hedonistic (PH), and Future (F). Four-week test-retest reliabilities range from .70 to .80, indicating high stability [12]. We used the mean of the respective items for the subscale scores.

#### Reduced Morningness-Eveningness Questionnaire (rMEQ)

We measured the individual chronotype with the reduced version of the Morningness-Eveningness Questionnaire (rMEQ, [28]). This scale involves 5 items allowing to classify participants sensitively and reliably into Morning-, Evening- or Intermediate-type. The reduced scale can be seen as an amelioration of the original MEQ and its internal consistency was evaluated as high [28]. Total scores can range from 4 to 25, where lower scores (4 to 10) refer to Evening-type, high scores (19 to 25) are classified as Morning-type, and the remainder allocates to the Intermediate-type.

#### Big Five Inventory (BFI-10)

The short version of the Big Five Inventory (BFI-10, [29]) consists of ten items—two for each of the five subscales measuring the Big Five personality dimensions: agreeableness, conscientiousness, extraversion, neuroticism, and openness. The statements are all rated on the same Likert scale ranging from 1 = strongly disagree to 5 = agree strongly. Cronbach`s α are appropriate with mean α = .75. The scores remain respectably stable, as indicated by an eight-week test-retest reliability is .72 [29]. We calculated the mean on the two items for the subscales, respectively.

### Statistical analyses

First, we analyzed differences in anxiety and depression concerning age, sex, and country. For this purpose, we ran general linear model analyses with anxiety and depression as dependent variable, respectively, with age as covariate, and sex and country as factors. Further, we computed post-hoc analyses, with Bonferroni correction, in order to assess differences between countries. Next, for each of the five ZTPI subscales as the dependent variable, we conducted general linear model analyses including age as covariate, and sex and country as factors. In this case, again, we computed post-hoc analyses with Bonferroni correction to test for differences between countries.

In order to extract the explanatory variables out of the set of proposed predictors that best predicted levels of anxiety and depression, respectively, we ran stepwise regression analyses (also referred to as fast stepwise procedure, FSP, [30, 31]). In contrast to hierarchical regression, the stepwise method uses an automatic procedure for the choice of predictive variables: Beginning from a null model (without predictors), in each step, the procedure both checks whether to add another predictive variable and whether to remove a previously added variable from the model that is not any more adding incremental validity to the prediction (unique predictive power; [32]). The feature of removing variables that are no longer of additional value makes this method superior to hierarchical regression modeling which is susceptible to the problem of multicollinearity. The model resulting from stepwise regression is similar to a best-subset regression where the combination of variables with the most explanatory power is selected out of all possible combinations [33], but is computationally less demanding. R^2^ values have been criticized to be usually overestimated with this method, therefore we will report a conservative adjusted R^2^ as an indicator of explained variance in the response variable (level of anxiety, level of depression). This conservative R^2^ is adjusted for the number of candidate predictors (rather than the number of selected predictors in the final model as usually done for adjustment) and provides a nearly unbiased estimate of R^2^ [34]:

$${conservative\;adj.\;R}^{2}=1-(1-R^{2})\frac{n-1}{n-k-1}\;with\;\begin{array}{c} n=sample\;size\\ k=number\;of\;candidate\;predictors \end{array}$$

For model selection, we resorted to the minimization of the Akaike Information Criterion (AIC; [35]). The AIC is a function of the prediction error of the model on the given data and includes a penalty term for the total number of predictors. A lower AIC corresponds to better goodness of fit under consideration of model parsimony. The AIC is thus an estimator of model quality. Once adding or removing further predictors does not reduce AIC, the algorithm stops.

In a first analysis, we entered the five time perspective subscales as well as age and sex into the saturated set of variables to be considered. In a subsequent analysis, we additionally included the Big-5 personality trait scales and the rMEQ. When predictor variables are highly correlated, only the variable with more explanatory power next to the other variables included will be extracted for the final model. This is desired in our study since we aim to explore the value of time perspectives as explanatory variables compared to the Big-5 personality traits.

Before computing the stepwise regression models, we computed the variance inflation factors (VIF) for all variables in all countries. The VIF is a quantification of multicollinearity in multiple regression models [36]. The square root of the VIF measures how much the variance of a regression coefficient might be increased due to multicollinearity [37]. With a conservative VIF < 3, multicollinearity is not a problem in the model [36]. VIF is computed as follows (with R^2^ representing the adjusted R^2^ when all other predictor variables included in the multiple regression model predict the remaining predictor variable):

$$VIF=\frac{1}{1-R^{2}}$$

All analyses were computed under a two-tailed significance level of *α* = .05 using the freeware statistics program R (https://www.r-project.org) and the GAMLj module for jamovi [38].

## Results

Descriptive statistics for the variables used in the present study are provided in Table 3.

**Table 3:** Descriptive statistics of the study variables for the whole sample and separately for each country.

	Argentina	France	Greece	Italy	Japan	Turkey	TOTAL
Sample Size (*n*)	206	474	173	170	87	171	1,294
Sex: F (% of *n*)	142 (70.40%)	340 (71.73%)	130 (75.14%)	108 (63.53%)	47 (54.02%)	132 (77.19%)	911 (70.40%)
Age M (SD)	33.95 (10.22)	40.87 (11.79)	30.87 (10.15)	26.66 (9.90)	40.34 (11.38)	22.77 (4.09)	34.19 (12.70)
HADS-Anxiety M (SD)	7.84 (4.24)	7.50 (4.04)	6.62 (4.08)	7.79 (4.33)	5.71 (3.33)	9.29 (4.41)	7.58 (4.21)
HADS-Depression M (SD)	6.52 (3.90)	5.53 (3.93)	6.24 (3.88)	4.96 (3.28)	9.12 (3.65)	7.92 (3.65)	6.27 (3.95)
ZTPI-Past Negative M (SD)	2.79 (0.76)	2.71 (0.82)	2.86 (0.79)	3.01 (0.77)	3.00 (0.78)	3.12 (0.72)	2.86 (0.80)
ZTPI-Past Positive M (SD)	3.54 (0.57)	3.31 (0.66)	3.44 (0.65)	3.45 (0.57)	3.33 (0.67)	3.60 (0.66)	3.42 (0.65)
ZTPI-Present Fatalistic M (SD)	2.38 (0.55)	2.45 (0.62)	2.42 (0.56)	2.45 (0.50)	2.75 (0.55)	2.56 (0.57)	2.47 (0.58)
ZTPI-Present Hedonistic M (SD)	3.05 (0.53)	3.36 (0.58)	3.39 (0.51)	3.32 (0.43)	3.07 (0.52)	3.37 (0.52)	3.29 (0.55)
ZTPI-Future M (SD)	3.47 (0.55)	3.49 (0.53)	3.62 (0.57)	3.66 (0.52)	3.58 (0.54)	3.55 (0.55)	3.54 (0.55)
BFI-Agreeableness M (SD)	3.33 (0.86)	2.45 (0.66)	3.37 (0.59)	3.16 (0.89)	2.83 (0.69)	3.26 (0.66)	3.04 (0.88)
BFI-Conscientiousness M (SD)	3.91 (0.91)	3.46 (0.66)	3.13 (0.63)	3.63 (0.87)	3.41 (0.66)	3.16 (0.61)	3.48 (0.77)
BFI-Extraversion M (SD)	2.81 (1.11)	3.27 (0.60)	3.33 (0.61)	3.22 (0.94)	2.98 (0.39)	3.14 (0.47)	3.16 (0.76)
BFI-Neuroticism M (SD)	3.23 (1.16)	3.23 (0.57)	3.29 (0.44)	3.10 (1.06)	3.24 (0.65)	3.16 (0.61)	3.21 (0.77)
BFI-Openness M (SD)	3.51 (0.84)	3.06 (0.79)	3.70 (0.87)	3.63 (0.98)	2.98 (0.62)	3.12 (0.66)	3.30 (0.86)
rMEQ M (SD)	13.91 (4.50)	15.81 (3.92)	13.08 (3.66)	14.39 (3.55)	14.93 (3.87)	11.82 (4.09)	14.35 (4.20)

### Effects of country, age, and sex on anxiety

**Fig 1:**
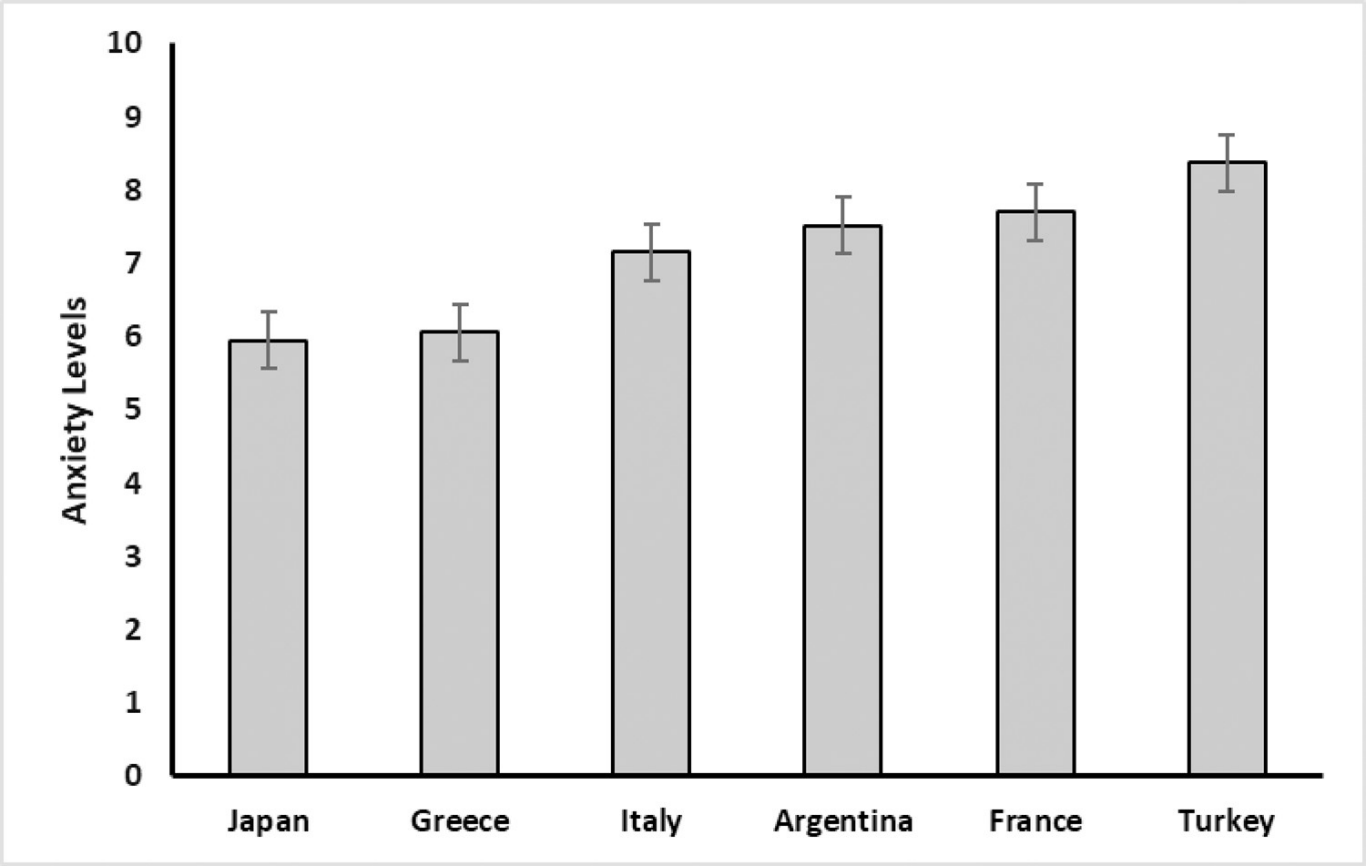
Average anxiety levels in the six studied countries. Whiskers represent the SE of the mean.

In order to test differences concerning anxiety levels between countries and sex we ran a general linear model (GLM) analysis, including age as covariate. A significant effect of age on anxiety was observed, *F*(1, 1,261) = 15.38, *p* < .*001*. Generally, younger participants reported higher levels of anxiety. There was also a significant effect of sex on anxiety levels, *F*(1, 1,261) = 27.55, *p* < .001. Females reported higher levels of anxiety when compared with males, *t*(1,261) = 5.25, *p*_*Bonferroni*_ < .001. The effect of country on anxiety was also significant, *F*(5, 1,261) = 6.87, *p* < .001. Specifically, there were significant pairwise differences between Argentina and Greece *t*(1,261) = 3.09, *p*_*Bonferroni*_ *=* .*030;* Greece and Turkey *t*(1,261) *=* -4.41, *p*_*Bonferroni*_ < .001; France and Greece *t*(1,261) = 3.83, *p*_*Bonferroni*_ < .002; France and Japan *t*(1,261) = 3.63, *p*_*Bonferroni*_ = .004; and Japan and Turkey *t*(1,261) = -3.99, *p*_*Bonferroni*_ = .001(see Fig 1).

### Effects of country, age, and sex on depression

**Fig 2:**
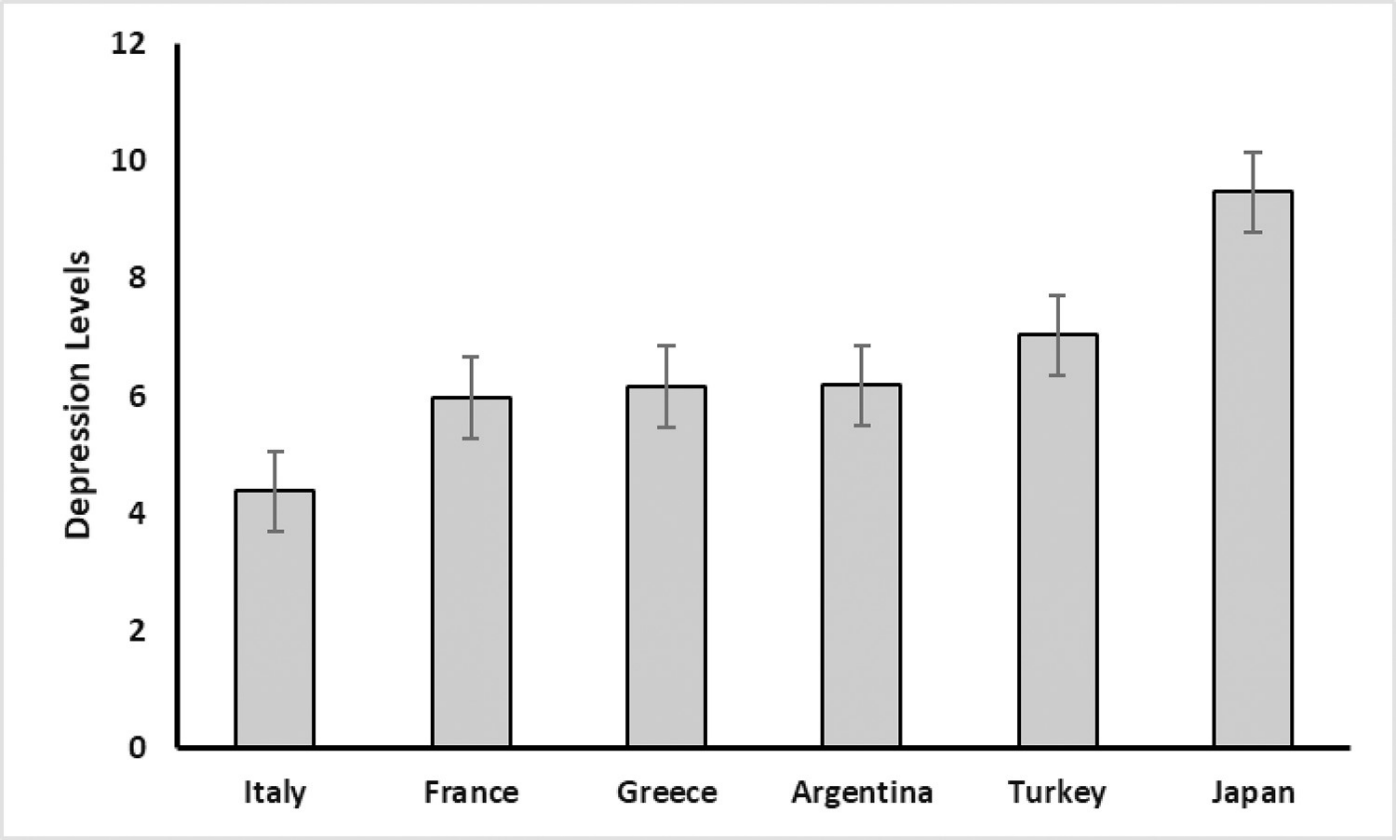
Average depression levels in the six studied countries. Whiskers represent the SE of the mean.

Next, with the same model but now with depression as the dependent variable, we again tested for differences between countries and sex with a GLM analysis, including age as a covariate. We observed a significant effect of age on depression, *F*(1, 1,261) = 30.93, *p* < .001, with younger participants reporting higher levels of depression compared to older individuals. There was also a significant effect of sex on depression, *F*(1, 1,261) = 5.24, *p* = .022. Again, females reported higher levels of depression than males, *t*(1,261) = 2.29, *p*_*Bonferroni*_ = .02. We also observed a statistically significant difference in levels of depression between countries, *F*(1, 1,261) = 21.96, *p* < .001. Italy reported significantly lower, and Japan exhibited significantly higher levels of depression compared to all other countries (see Fig 2). There were no significant differences between Argentina, France, Greece, and Turkey.

### Differences in time perspectives

**Fig 3:**
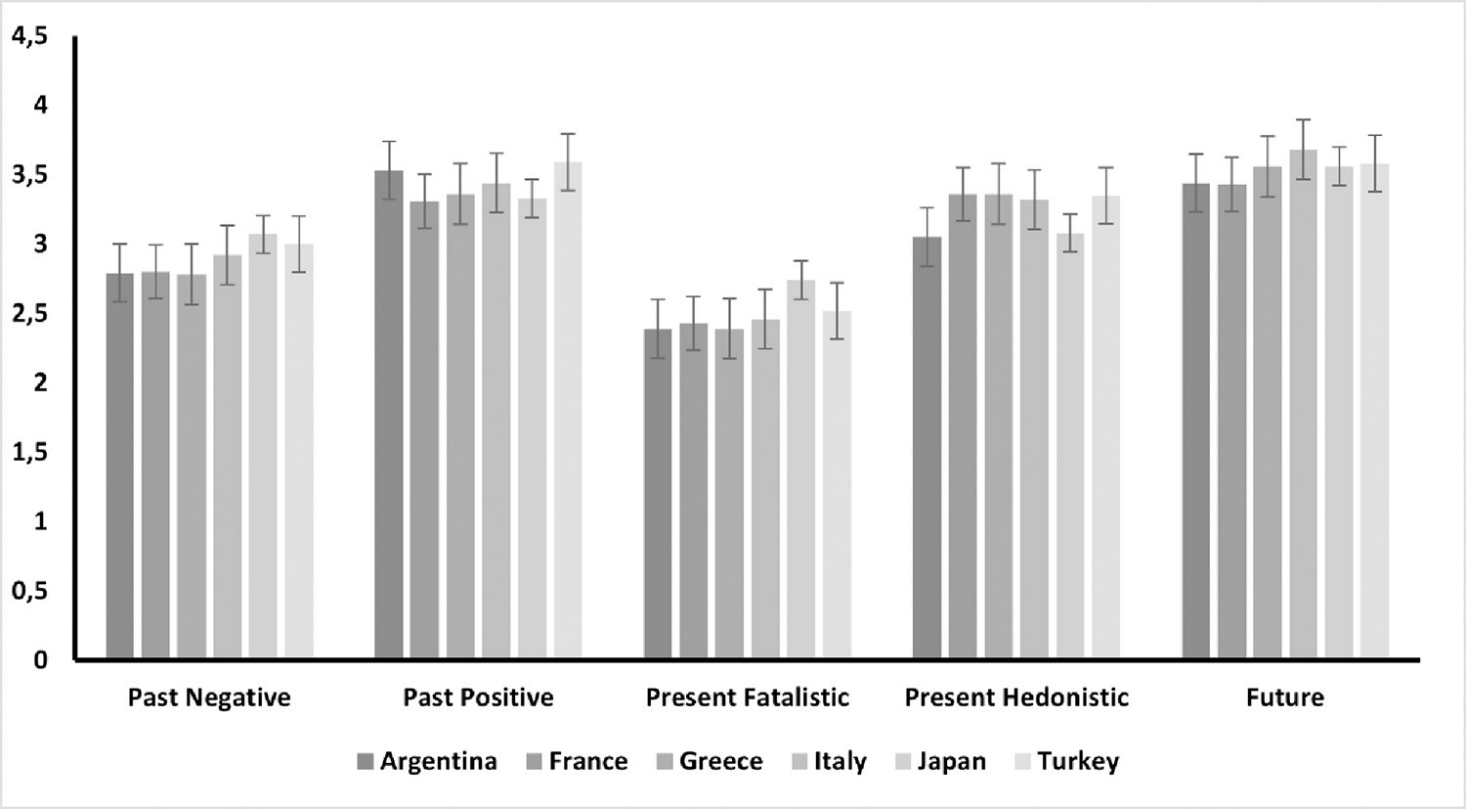
Average scores on the Zimbardo Time Perspectives Inventory subscales for each country.

For each of the Zimbardo Time Perspective Inventory subscales as a dependent variable, we computed a general linear model analysis, with age as a covariate, and sex and country as factors. A graphical overview of the average levels on each subscale by country is presented in Fig 3. For the Past Negative (PN) subscale, we observed a significant effect of age, *F*(1, 1,268) = 29.646, *p* < .001. Younger participants reported higher scores on the PN subscale, *t*(1,268) = -5.44, *p* < .001. Although we found a significant effect of country, *F*(1, 1,268) = 3.20, p = .007, none of these differences was found to be statistically significant in the post-hoc analyses. The effect of sex on the PN subscale was non-significant, *t(1*,*268) = -1*.*09*, *p =* .*275*.

On the Past Positive (PP) subscale, the model indicated a significant effect only for country, *F*(5,1,268) = 4.59, *p* < .001. The post-hoc analyses showed that there were statistically significant differences between French and Turkish participants, *t*(1,268) = -3.79, *p*_*Bonferroni*_ = .002, and between Argentinian and French participants, *t*(1,268) = 3.71, *p*_*Bonferroni*_ = .003. French participants scored lowest on the PP subscale on average. The effects of sex on *PP (F(1*, *1*,*268) = 3*.*05*, *p =* .*081)* and of age on PP *(F(1*, *1*,*268) = 0*.*176*, *p =* .*674)* were non-significant.

Concerning the Present Fatalistic (PF) subscale, we observed a significant effect of country, *F*(5, 1,268) = 5.39, *p* < .001. Japanese participants reported the higher levels of PF. The post-hoc analyses revealed significant differences between Argentinian and Japanese participants, *t*(1,268) = -4.59, *p*_*Bonferroni*_ < .001, Greek and Japanese participants, *t*(1,268) = -4.22, *p*_*Bonferroni*_ < .001, French and Japanese participants, *t*(1,268) = -4.51, *p*_*Bonferroni*_ < .001, and Italian and Japanese participants *t*(1,268) = -3.49, *p*_*Bonferroni*_ *=* .*007*. The effects of sex on PF (*F(1*, *1*,*268) = 2*.*91*, *p =* .*088*) and of age on PF (*F(1*, *1*,*268) = 0*.*55*, *p =* .*455*) were non-significant.

For the Present Hedonistic (PH) subscale, we observed a significant effect of country, *F*(5, 1,268) = 12.20, *p* < .001. Argentinian and Japanese participants reported the lowest scores of PH on average. In the post-hoc analyses, we found significant differences between Argentinian and Greek participants, *t*(1,268) = -5.10, *p*_*Bonferroni*_ < .001, Argentinian and French participants, *t*(1,268) = -6.31, *p*_*Bonferroni*_ < .001, Argentinian and Italian participants, *t*(1,268) = -4.66, *p*_*Bonferroni*_ < .001; Argentinian and Turkish participants, *t*(1,268) = -4.67, *p*_*Bonferroni*_ < .001, as well as between Greek and Japanese participants, *t*(1,268) = 3.78, *p*_*Bonferroni*_ *=* .002, French and Japanese participants, *t*(1,268) = 4.45, *p*_*Bonferroni*_ < .001, Japanese and Turkish participants, *t*(1,268) = -3.44, *p*_*Bonferroni*_ *=* .009, and Italian and Japanese participants, *t*(1,268) = 3.32, *p*_*Bonferroni*_ = .014. Again, effects of sex on PH (*F(1*, *1*,*268) = 0*.*31*, *p =* .*575*) and of age on PH (*F(1*, *1*,*268) = 0*.*97*, *p =* .*324*) were non-significant.

Finally, for the Future subscale we observed a significant effect of age, *F*(5, 1,268) = 5.14, *p* = .024. Younger participants reported lower scores on this subscale on average when compared to older individuals. A significant effect of sex was also found, *F*(5, 1,268) = 5.77, *p* = .016. Females reported higher levels on the Future Subscale compared to males, *t*(1,268) = 2.40, *p*_*Bonferroni*_ = .016. The effect of country was also significant, *F*(5, 1,268) = 5.00, *p* < .001. Italian participants reported higher scores in the future subscale compared to French and Argentinian participants: The post-hoc analysis showed significant differences between Argentinians and Italians, *t*(1,268) = -3.91, *p*_*Bonferroni*_ = .001, as well as French and Italian participants, *t*(1,268) = -4.43, *p*_*Bonferroni*_ < .001.

### Stepwise regression models

With stepwise regression models, we aimed to find the model that best and most parsimoniously predicted the levels of anxiety and depression, respectively, in each of the six countries included in our sample. First, we checked for multicollinearity in the multiple regression models using the VIF. The largest VIF was 1.68 (observed for Past Negative in Argentina). Following the interpretation rules, this value indicates no matter for concern and the subsequent regression analyses can be conducted [36].

### Stepwise regression of anxiety on personality traits

The detailed results of the stepwise regression models for the prediction of anxiety are shown in Table 4. First, we considered the five subscales of the ZTPI next to age and sex as potential predictors (ZTPI model). A negative attitude toward the past and present affected the levels of anxiety significantly in many countries. We found that the Past Negative subscale was a significant positive predictor in all countries. The scores on the Present Fatalistic subscale also significantly and positively predicted anxiety levels in all countries except for Argentina and Japan. For a positive attitude towards the past and present, the results were less consistent. In particular, we observed a significant positive effect of Present Hedonistic only in the Japanese sample whereas Past Positive had a significant negative effect in the Turkish sample only. The future subscale predicted anxiety levels positively in Italy and Turkey, but did not reach statistical significance in Argentina and France (both *p* < .10). Sex was negatively related to anxiety levels, indicating that males experience lower levels of anxiety than female in all countries except for France and Japan (where sex was not a significant predictor). Age negatively predicted anxiety levels in Italy only. The final models with age, sex, and the ZTPI subscales as potential predictors accounted for 12% (Japan) to 30% (Turkey) of the variance in anxiety levels (mean explained variance across countries = 23.43%, SD = 6.53%), as indicated by the conservatively adjusted R^2^ of the respective models.

**Table 4:** Final models for anxiety after stepwise regression (country-wise, with two models each (1^st^: ZTPI, sex, and age as predictors; 2^nd^ -extended: ZTPI, sex, age, BFI, and rMEQ as predictors)).

Country	Argentina (n = 206)		France (n = 449)		Greece (n = 169)		Italy (n = 170)		Japan (n = 87)		Turkey (n = 171)	
Scales	ZTPI	extended	ZTPI	extended	ZTPI	extended	ZTPI	extended	ZTPI	extended	ZTPI	extended
age							-0.08** (0.03)				-0.11^()^ (0.07)	
Sex (M)	-1.55** (0.57)	-0.87† (0.52)			-1.43* (0.64)	-1.43* (0.64)	-2.23*** (0.59)	-1.77** (0.55)		-1.29* (0.64)	-1.52* (0.69)	
Past Neg.	2.39*** (0.35)	1.29*** (0.35)	2.20*** (0.22)	2.22*** (0.22)	1.75*** (0.39)	1.75*** (0.39)	1.42*** (0.41)	0.80* (0.38)	1.21** (0.44)	0.77† (0.43)	2.10*** (0.45)	2.19*** (0.42)
Past Pos.					-0.69^()^ (0.44)	-0.69^()^ (0.44)					-1.39** (0.44)	-1.32** (0.43)
Pres. Fatal.			0.69* (0.30)	0.76* (0.30)	1.84*** (0.52)	1.84*** (0.52)	2.47*** (0.67)	1.49* (0.60)			1.35* (0.55)	1.79** (0.56)
Pres. Hedon.									1.68* (0.65)	1.53* (0.62)		
Future	0.83† (0.48)	0.71^()^ (0.43)	0.61† (0.32)	0.58† (0.32)			1.41* (0.56)	1.20* (0.51)			1.35* (0.54)	1.50** (0.53)
Agreeablen.												-0.83† (0.43)
Conscient.												
Extraversion				-0.46^()^ (0.28)						2.27** (0.84)		0.86^()^ (0.59)
Neuroticism		1.62*** (0.23)		-0.56† (0.29)				1.64*** (0.27)		-1.31* (0.50)		1.01* (0.46)
Openness								0.75** (0.27)				-1.10* (0.45)
rMEQ								-0.15* (0.07)				
adj. *R*^*2*^	.222	.370	.239	.247	.299	.299	.270	.421	.173	.266	.304	.336
cons. adj. R^2^	.206	.340	.232	.233	.286	.259	.261	.398	.121	.185	.300	.315
RSS	2831.40	2282.70	5625.10	5539.10	1958.20	1958.20	2263.70	1774.10	778.38	666.53	2236.7	2108.4
*F*	48.32***		3.44*		---		22.35***		4.53**		4.93**	
*df*	202	201	445	443	164	164	164	162	84	81	164	162

*Note*. If not otherwise indicated, unstandardized estimates (b-values) are reported with standard errors in brackets. The F-test compares the models within each country (ZTPI, sex, and age vs. ZTPI, sex, age, BFI, rMEQ).

***p < .001

**p < .01

*p < .05

†*p* < .10, n.s.; ^()^ *p* > .10, n.s.; adj. R2 = adjusted *R*^*2*^; cons. adj. R2 = conservative adjusted *R*^*2*^ (cf. Harrell, 2001); RSS = residual sum of squares; *F* = *F* test statistic; *df* = degrees of freedom; *p* = *p*-value.

Next, we also considered the BFI and the rMEQ as additional potential predictors next to the ZTPI, age, and sex (extended model). Neuroticism was retained in the final models in all countries except Greece. However, whereas neuroticism predicted anxiety levels positively in Argentina, Italy, and Turkey, it was a negative predictor in Japan. In Japan, extraversion positively predicted anxiety levels. Openness predicted anxiety positively in Italy, and negatively in Turkey. Italy was furthermore the only country in which the chronotype as measured with the rMEQ (negatively) predicted anxiety levels. That is, individuals with pronounced eveningness are more likely to report higher anxiety levels in this first phase of the pandemic. With the added predictors, the ZTPI subscales retained in the first (ZTPI) model were also retained in the extended model. Only in Japan, the PN subscale was rendered non-significant (*p* < .10), but still remained in the final model. Sex was no longer retained as a predictor in Turkey, age was no longer retained in Italy, and in Argentina, sex remained in the model but was no longer significant (*p* < .10). With the additional predictors (BFI, rMEQ), the percentage of explained variance in anxiety levels was raised considerably in Argentina (from 21% to 34%) and in Italy (from 26% to 40%), and less strongly in Japan (from 12% to 19%) and in Turkey (from 30% to 32%), as indicated by the conservatively adjusted R^2^ of the respective final models. In France, the R^2^ stayed the same (.23), in Greece the value decreased slightly (due to the penalty for the included potential predictors) from .29 to .26. The mean explained variance across countries was 28.83% (SD = 7.74%) for the extended model (conservatively adjusted R^2^).

### Stepwise regression of depression on personality traits

For the depression models, the detailed statistics of the final models after the stepwise regression are presented in Table 5. The results for the ZTPI model (including ZTPI, age, and sex as potential predictors) indicated that the Past Negative subscale consistently positively predicted depression levels in all countries except for Japan where PN was not retained in the model. Past Positive was a significant negative predictor in the model in all countries except for Italy and Argentina (*p* < .10). The Present Fatalistic subscale positively predicted depression in France and Italy. Positive attitudes to the present (Present Hedonistic subscale) negatively predicted depression levels in France. The Future subscale acted as a significant negative predictor in Japan only. Age was negatively associated with reported depression symptoms in France, Italy, and Turkey. Sex predicted depression scores on the HADS in Argentina and Italy, where being female was an indicator for higher levels of depression. The final ZTPI models with age, sex, and the ZTPI subscales as potential predictors accounted for 14% (Japan) to approximately 20% (Greece, Italy, and Turkey) of the variance in depression levels (mean explained variance across countries = 17.90%, SD = 2.45%), as indicated by the conservatively adjusted R^2^ of the respective models.

**Table 5:** Final models for depression after stepwise regression (country-wise, with two models each (1^st^: ZTPI, sex, and age as predictors; 2^nd^ = extended: ZTPI, sex, age, BFI, and rMEQ as predictors)).

Country	Argentina (n = 206)		France (n = 449)		Greece (n = 169)		Italy (n = 170)		Japan (n = 87)		Turkey (n = 171)	
Scales	ZTPI	extended	ZTPI	extended	ZTPI	extended	ZTPI	extended	ZTPI	extended	ZTPI	extended
Age	-0.04† (0.02)		-0.04** (0.01)	-0.04** (0.01)			-0.06* (0.02)	-0.06* (0.02)			-0.16* (0.06)	-0.11† (0.07)
Sex (M)	-1.82*** (0.52)	-1.67** (0.53)					-1.08* (0.47)	-1.10* (0.48)			-0.99^()^ (0.61)	-0.91^()^ (0.60)
Past Neg.	1.77*** (0.32)	1.12** (0.36)	1.28*** (0.24)	1.28*** (0.24)	1.56*** (0.39)	1.53*** (0.39)	1.14*** (0.33)	1.10** (0.33)			1.07** (0.39)	1.16** (0.38)
Past Pos.	-0.74† (0.42)	-0.89* (0.41)	-0.62* (0.26)	-0.62* (0.26)	-1.24** (0.43)	-1.49*** (0.43)			-1.20* (0.55)	-1.20* (0.55)	-1.43*** (0.38)	-1.37*** (0.38)
Pres. Fatal.			1.12*** (0.32)	1.12*** (0.32)	0.92† (0.53)	1.16* (0.52)	1.50** (0.54)	1.28* (0.55)			0.87† (0.47)	0.90† (0.46)
Pres. Hedon.			-0.82* (0.32)	-0.82* (0.32)			-0.94† (0.54)	-1.04† (0.55)				
Future									-2.34*** (0.68)	-2.34*** (0.68)		
Agreeablen.												-0.93* (0.38)
Conscient.												
Extraversion						0.79† (0.44)		0.62* (0.25)				
Neuroticism		0.98*** (0.23)						0.40† (0.24)				
Openness		-0.57† (0.29)				-0.72* (0.31)						
rMEQ						-0.13† (0.07)						-0.08^()^ (0.06)
adj. *R*^*2*^	.197	.257	.181	.181	.218	.263	.202	.234	.188	.188	.212	.238
cons. adj. R^2^	.169	.226	.177	.166	.198	.230	.192	.205	.136	.065	.202	.209
RSS	2508.70	2266.90	5650.80	5650.80	1988.3	1838.5	1417.80	1343.40	921.52	921.52	1738.20	1660.50
*F*	10.67***		---		4.40**		4.49*		---		3.81*	
*df*	202	200	443	443	165	162	164	162	84	84	165	163

*Note*. If not otherwise indicated, unstandardized estimates (b-values) are reported with standard errors in brackets. The F-test compares the models within each country (ZTPI, sex, and age vs. ZTPI, sex, age, BFI, rMEQ).

***p < .001

**p < .01

*p < .05

†*p* < .10, n.s.; ^()^ *p* > .10, n.s.; adj. R2 = adjusted *R*^*2*^; cons. adj. R2 = conservative adjusted *R*^*2*^ (cf. Harrell, 2001); RSS = residual sum of squares; *F* = *F* test statistic; *df* = degrees of freedom; *p* = *p*-value.

Next, for the extended model, we additionally included the BFI and the rMEQ as additional potential predictors next to the ZTPI, age, and sex. There was no consistent pattern of these predictors. Agreeableness negatively predicted depression in Turkey only, whereas extraversion was a positive predictor in Italy. Neuroticism positively predicted depression levels in Argentina. Openness was a negative predictor in Greece. The rMEQ was retained in the final models as a negative predictor for depression in the Greek and Turkish sample, but both times was not a significant predictor itself. All ZTPI subscales remained in the model when the additional predictors were included; Past Positive in Argentina and Present Fatalistic in Greece became significant predictors (after having been non-significant with *p* < .10 in the model with ZTPI, sex, and age as predictors). Age was no longer retained as a significant negative predictor in Argentina and Turkey. For sex, there were no differences compared to the first (ZTPI) model. With the added predictors (BFI, rMEQ), the percentage of variance in depression levels accounted for by the final models increased in Argentina (from 17% to 23%) and in Greece (from 20% to 23%). There were also slight increases in the conservatively adjusted R^2^ in Italy (from .19 to .21), and in Turkey (from .20 to .21). The R^2^ decreased slightly in France (from .18 to .17), and strongly in Japan (from .14 to .07) because of the penalty for the included potential predictors. The mean explained variance of scores on the HADS depression subscale across countries was 18.35% (SD = .45%) for this extended model.

## Discussion

The aim of this study was to assess personality traits and psychological wellbeing of people from six countries during the COVID-19 pandemic. More specifically, we aimed at giving a snapshot of the levels of anxiety and depression during the first weeks of the pandemic in spring 2020, looking at differences between countries, ages, and sex. We found that there were significant differences between countries. For instance, Japanese and Greek participants reported low levels of anxiety whereas Turkish exhibited the highest scores; and on the HADS depression subscale, Italian participants reported the lowest levels while Japanese scored highest.

Our results are in line with the meta-analysis conducted by Salari and colleagues [3], who reported that women and young people experienced higher levels of depression and anxiety (than men and older individuals) during the COVID-19 outbreak. Over the whole sample and across countries, we found a significant effect of sex on both anxiety and depression showing that females reported higher levels of anxiety and depression than males. Additionally, we found that irrespective of country, the younger the participants, the higher they scored on both anxiety and depression scales.

Furthermore, we analyzed whether and to what extent relatively time-stable personality traits of time perspectives, chronotype, and the Big Five could predict the reported levels of anxiety and depression. The literature reports an association of time perspectives with psychological well-being [12, 18]. Similarly, our results consistently showed effects of the Past Negative subscale on anxiety in all the tested countries (Argentina, France, Greece, Italy, Japan, and Turkey) and depression in all countries except Japan. A more negative vision of the past, characterized by negative memories and regrets, seems to be predictive of increased symptoms of both anxiety and depression during confinement. The Present Fatalistic subscale also predicted higher levels of depression and anxiety in many countries but was not as consistent a predictor as Past Negative. Instead, for depression, Past Positive was a negative predictor for many countries (except for Italy). Additionally, the Present Hedonistic factor negatively predicted depression in France only and positively predicted anxiety in the Japanese sample only. Future orientation was a significant negative predictor for depression in Japan, and positively predicted anxiety in Italy and Turkey. Importantly, the time perspective subscales were retained as predictors once the Big Five and rMEQ (chronotype) were added as potential predictors in the extended model.

Interestingly, Japan showed a pattern different from all the other countries. Next to the Past Negative factor as also commonly observed in other countries, the Present Hedonistic perspective was a predictor of higher levels of anxiety. Sex, Extraversion, and Neuroticism added explanatory power to the model for anxiety. Past Positive and Future orientation both negatively predicted depression and were the only relevant variables retained in the final model. At the same time, the Japanese sample showed the highest levels of depression and the lowest levels of anxiety, a pattern that differs from other countries where the levels of anxiety was always higher—at least, nominally–than the level of depression. What are the possible reasons for these differing results with the Japanese sample compared to the other countries? Next to possible (speculative) cultural explanations, the Japanese sample differs from the other countries in that it is much smaller than the others, participants were paid for partaking in the study, the sample’s age was more evenly distributed (similar to France), and it is the only country where there was no strict lockdown (as reflected in the mobility and stringency indices in Table 2). Of note, in line with our results, other studies conducted during the pandemic period in Japan showed more pronounced levels of depression than anxiety [39, 40], with mean HADS values similar to the ones reported in the present study [41].

The Big Five Inventory did not contribute in a consistent way to the final regression models; however, some of the factors were retained in some of the countries. Neuroticism was included in the final model for anxiety in most of the countries, but the direction of the effect was not consistent: neuroticism was a positive predictor in Argentina, Italy, and Turkey, whereas it negatively predicted anxiety in Japan. Nevertheless, the Big Five scales added explanatory power to the models for anxiety (considerably in Argentina, Italy, Japan, but just a bit in Turkey) and for depression (considerably in Argentina, but just a bit in Greece, Italy, and Turkey) even when considering the conservative adjusted R^2^. Generally, both the model with ZTPI, sex, and age and the extended model with additional predictors, BFI and, accounted for more variance in the models predicting the scores on the HADS anxiety subscale than those predicting scores on the depression subscale.

Although previous literature reported relationships between evening orientation and the levels of anxiety and depression, chronotype was not retained in the models in conjunction with the other personality traits (except for the Italian sample where eveningness additionally predicted higher levels of anxiety). These results can be explained considering that the social jetlag [42] may have been reduced during the pandemic inindividuals who experienced a lockdown with strict restrictions [6, 43–45].

The current study has some limitations. First, the general study in which the questionnaires were included required a strong commitment of participants since the project comprised multiple long sessions of three to four hours. This might result in a non-representativeness of samples. Note that Japanese participants were the only paid subjects and the age distribution was even in Japan and France, but not in the other countries where mostly young people participated. Second, sex was also not balanced, with proportions of female participation varying from 54% in Japan to 75% in Greece. Third, our interpretation of our results is limited to the data we collected. For example, information about the employment and economic condition of our participants, as well as their family situation (e.g, having a close family member who is sickcaring for young children who could not attend school or daycare in presence) could have been very useful in the interpretation of the data.

## Conclusions

In summary, the present study provides data about anxiety and depression during the pandemic and compares them in a variety of countries. Time perspectives as derived from the ZTPI are good predictors for anxiety and depression with explained variance for anxiety between 12% (Japan) and 30% (Turkey), and between 17% (Argentina) and 21% (Turkey) for depression. Particularly, Past Negative was a very consistent, and Present Fatalistic a little bit less consistent predictor for both anxiety and depression. The Past Positive subscale negatively predicted depression in most countries. Importantly, the ZTPI subscales remain significant predictors also when the Big Five and chronotype are added to the models. The Big Five can add explanatory power to the regression models in some cases, but we observed no consistent predictors for either anxiety or depression. Neuroticism was the only factor that was retained in most countries for anxiety, but sometimes the association was positive (AR, IT, TR), sometimes negative (FR, JP). Chronotype (rMEQ) generally did not benefit the regression models. The present study suggests that the orientation towards time might be valuable as proxies for the individual risk of exhibiting heightened levels of depression and anxiety symptoms during the pandemic.

## References

[1] Galea S., Merchant R. M., & Lurie N. (2020). The mental health consequences of COVID-19 and physical distancing: the need for prevention and early intervention. *JAMA Internal Medicine*, 180(6), 817–818. doi: 10.1001/jamainternmed.2020.1562 32275292

[2] Brooks S. K., Webster R. K., Smith L. E., Woodland L., Wessely S., Greenberg N., et al. (2020). The psychological impact of quarantine and how to reduce it: rapid review of the evidence. *The lancet*, 395(10227), 912–920. 10.1016/S0140-6736(20)30460-8 PMC7158942 32112714

[3] Salari N., Hosseinian-Far A., Jalali R., Vaisi-Raygani A., Rasoulpoor S., Mohammadi M., et al. (2020). Prevalence of stress, anxiety, depression among the general population during the COVID-19 pandemic: a systematic review and meta-analysis. *Globalization and health*, 16(1), 1–11. 32631403 10.1186/s12992-020-00589-w PMC7338126

[4] Grondin S., Mendoza-Duran E., & Rioux P. A. (2020). Pandemic, Quarantine, and Psychological Time. *Frontiers in Psychology*, 11. 10.3389/fpsyg.2020.581036 PMC7641621 33192897

[5] Wang C., Pan R., Wan X., Tan Y., Xu L., Ho C. S., et al. (2020). Immediate Psychological Responses and Associated Factors during the Initial Stage of the 2019 Coronavirus Disease (COVID-19) Epidemic among the General Population in China. *International Journal of Environmental Research and Public Health*, 17(5), 1729. doi: 10.3390/ijerph17051729 32155789 PMC7084952

[6] Cellini N., Canale N., Mioni G., & Costa S. (2020). Changes in sleep pattern, sense of time and digital media use during COVID‐19 lockdown in Italy. *Journal of Sleep Research*, 29(4), e13074. doi: 10.1111/jsr.13074 32410272 PMC7235482

[7] Ogden R. S. (2020). The passage of time during the UK Covid-19 lockdown. *PLoS ONE*, 15(7), e0235871. doi: 10.1371/journal.pone.0235871 32628735 PMC7337311

[8] Droit-Volet S., S, Gil S., Martinelli N., Andant N., Clinchamps M., Parreira L., et al. (2020) Time and Covid-19 stress in the lockdown situation: Time free, «Dying» of boredom and sadness. *PLoS ONE*, 15(8): e0236465. doi: 10.1371/journal.pone.0236465 32776990 PMC7416923

[9] Wittmann M. (2020). Subjective Passage of Time during the Pandemic: Routine, Boredom, and Memory. *KronoScope*, 20(2), 260–271.

[10] Avni-Babad D., & Ritov I. (2003). Routine and the perception of time. *Journal of Experimental Psychology*: *General*, 132(4), 543. doi: 10.1037/0096-3445.132.4.543 14640847

[11] Kairys A., & Liniauskaite A. (2015). Time Perspective and Personality. In: Stolarski M., Fieulaine N., & Beek W. (Eds.), *Time perspective theory; review*, *research and application* (pp. 99–113). Springer, Cham.

[12] Zimbardo P. G., & Boyd J. N. (1999). Putting time in perspective: A valid, reliable individual-differences metric. In *Time perspective theory*; *review*, *research and application* (pp. 17–55). Springer, Cham.

[13] Jochemczyk Ł, Pietrzak J., Buczkowski R., Stolarski M., & Markiewicz Ł (2017). You Only Live Once: Present-hedonistic time perspective predicts risk propensity. *Personality and Individual Differences*, 115, 148–153. 10.1016/j.paid.2016.03.010

[14] Stolarski M., Zajenkowski M., Zajenkowska A. (2016). Aggressive? from Time to Time… Uncovering the Complex Associations between Time Perspectives and Aggression. *Current Psychology*, 35, 506–515. 10.1007/s12144-016-9422-6

[15] Zimbardo P., & Boyd J. (2008). *The time paradox*: *The new psychology of time that will change your life*. Simon and Schuster.

[16] Sircova A., Van De Vijver F. J., Osin E., Milfont T. L., Fieulaine N., Kislali-Erginbilgic A., et al. (2014). A global look at time: A 24-country study of the equivalence of the Zimbardo Time Perspective Inventory. *Sage Open*, 4(1), 2158244013515686.

[17] Sircova A., Van De Vijver F. J., Osin E., Milfont T. L., Fieulaine N., Kislali-Erginbilgic A., et al. (2015). Time perspective profiles of cultures. In: Stolarski M., Fieulaine N., & Beek W. (Eds.), *Time perspective theory; review*, *research and application* (pp. 169–187). Springer, Cham.

[18] Desmyter F., & De Raedt R. (2012). The relationship between time perspective and subjective well-being of older adults. *Psychologica Belgica*, 52(1), 19–38.

[19] Papastamatelou J., Unger A., Giotakos O., & Athanasiadou F. (2015). Is time perspective a predictor of anxiety and perceived stress? Some preliminary results from Greece. *Psychological Studies*, 60(4), 468–477.

[20] Cox R. C., & Olatunji B. O. (2019). Differential associations between chronotype, anxiety, and negative affect: A structural equation modeling approach. *Journal of affective disorders*, 257, 321–330. doi: 10.1016/j.jad.2019.07.012 31302521 PMC6711779

[21] Kivelä L., Papadopoulos M. R., & Antypa N. (2018). Chronotype and psychiatric disorders. *Current sleep medicine reports*, 4(2), 94–103. doi: 10.1007/s40675-018-0113-8 29888167 PMC5972175

[22] Merikanto I., Kronholm E., Peltonen M., Laatikainen T., Vartiainen E., & Partonen T. (2015). Circadian preference links to depression in general adult population. *Journal of Affective Disorders*, 188, 143–148. doi: 10.1016/j.jad.2015.08.061 26363264

[23] Kotov R., Gamez W., Schmidt F., & Watson D. (2010). Linking “big” personality traits to anxiety, depressive, and substance use disorders: a meta-analysis. *Psychological bulletin*, 136(5), 768. doi: 10.1037/a0020327 20804236

[24] van Wassenhove V., Balcı F., Giersch A., Gronfier C., & Vatakis A. (2020, August 28). *TimeSocialDistancing*. 10.17605/OSF.IO/9E324

[25] Hale T., Angrist N., Goldszmidt R., Kira B., Petherick A., Phillips T., et al. (2021). A global panel database of pandemic policies (Oxford COVID-19 Government Response Tracker). *Nature Human Behaviour*, 5, 529–538. doi: 10.1038/s41562-021-01079-8 33686204

[26] Zigmond A. S., & Snaith R.P. (1983). The Hospital Anxiety and Depression Scale. *Acta Psychiatrica Scandinavica*, 67,361–370. doi: 10.1111/j.1600-0447.1983.tb09716.x 6880820

[27] Crawford J.R., Henry J. D., Crombie C., & Taylor E. P. (2001). Normative data for the HADS from a large non-clinical sample. *British Journal of Clinical Psychology*, 40, 429–434. doi: 10.1348/014466501163904 11760618

[28] Adan A. & Almirall H. (1991). Horne & Östberg Morningness-Eveningness Questionnaire: A Reduced Scale. *Personality and Individual Differences*, 12(3), 241–253.

[29] Rammsted B. & John O. P. (2007). Measuring personality in one minute or less: A 10-item short version of the Big Five Inventory in English and German. *Journal of Research in Personality*, 41, 203–212.

[30] An H., & Gu L. (1989). Fast Stepwise Procedure of Selection of Variables by Using AIC and BIC Criteria. *ACTA Mathematicae Applicatae Sinica*, 5(1), 60–67.

[31] Yamashita T., Yamashita K., & Kamimura R. (2007) A Stepwise AIC Method for Variable Selection in Linear Regression, Communications in Statistics—Theory and Methods, 36:13, 2395–2403, doi: 10.1080/03610920701215639

[32] Thompson B. (1989). Why won’t stepwise methods die? Measurement and Evaluation in Counseling and Development, 21(4), 146–148.

[33] Ruengvirayudh P., & Brooks G. P. (2016). Comparing Stepwise Regression Models to the Best-Subsets Models, or, the Art of Stepwise. *General Linear Model Journal*, 42(1), 1–14.

[34] Harrell F. E. (2001). *Regression modeling strategies*: *with applications to linear models*, *logistic regression*, *and survival analysis*. Springer.

[35] Akaike H. (1974). A new look at the statistical model identification. *IEEE transactions on automatic control*, 19(6), 716–723.

[36] Hair J.F., Risher J.J., Sarstedt M., & Ringle C.M. (2019). When to use and how to report the results of PLS-SEM. *European Business Review*, 31(1), 2–24. 10.1108/EBR-11-2018-0203

[37] Fox J. (2005). Linear Models, Problems. In Kempf-Leonard K. (Ed.), *Encyclopedia of Social Measurement* (pp.515–522). Cambridge, MA, USA: Elsevier Academic Press.

[38] Gallucci M. (2019). *GAMLj*: *General analyses for linear models*. [jamovi module]. Retrieved from https://gamlj.github.io/

[39] Stickley A., Matsubayashi T., Sueki H., & Ueda M. (2020). COVID-19 preventive behaviours among people with anxiety and depressive symptoms: findings from Japan. *Public Health*, 189, 91–93 doi: 10.1016/j.puhe.2020.09.017 33189941 PMC7547627

[40] Tatsuno J., Unoki T., Sakuramoto H., & Hamamoto M. (2021). Effects of social support on mental health for critical care nurses during the coronavirus disease 2019 (COVID‐19) pandemic in Japan: A web‐based cross‐sectional study. *Acute Medicine & Surgery*, 8(1), e645 doi: 10.1002/ams2.645 33868689 PMC8035953

[41] Wakashima K., Asai K., Kobayashi D., Koiwa K., Kamoshida S., & Sakuraba M. (2020). The Japanese version of the Fear of COVID-19 scale: Reliability, validity, and relation to coping behavior. *PloS one*, 15(11), e0241958 doi: 10.1371/journal.pone.0241958 33152038 PMC7644080

[42] Wittmann M., Dinich J., Merrow M., & Roenneberg T. (2006). Social jetlag: misalignment of biological and social time. *Chronobiology International*, 23(1–2), 497–509. doi: 10.1080/07420520500545979 16687322

[43] Blume et al. 2020. Effects of the COVID-19 lockdown on human sleep and rest-activity rhythms. *Current Biology*, 30(14), R795–R797. doi: 10.1016/j.cub.2020.06.021 32693067 PMC7284244

[44] Leone M. J., Sigman M., & Golombek D. A. (2020). Effects of lockdown on human sleep and chronotype during the COVID-19 pandemic. *Current Biology*, 30(16), R930–R931. doi: 10.1016/j.cub.2020.07.015 32810450 PMC7342078

[45] Wright K. P. Jr, Linton S. K., Withrow D., Casiraghi L., Lanza S. M., de la Iglesia H., et al. (2020). Sleep in university students prior to and during COVID-19 Stay-at-Home orders. *Current Biology*, 30(14), R797–R798. doi: 10.1016/j.cub.2020.06.022 32693068 PMC7284257

