# Correction: Time perspective predicts levels of anxiety and depression during the COVID-19 outbreak: A cross-cultural study

**DOI:** 10.1371/journal.pone.0314047

**Published:** 2024-11-13

**Authors:** Luigi Micillo, Pier-Alexandre Rioux, Esteban Mendoza, Sebastian L. Kübel, Nicola Cellini, Virginie Van Wassenhove, Simon Grondin, Giovanna Mioni

The first two authors, Luigi Micillo and Pier-Alexandre Rioux, should not have been attributed equal contribution to this work. Instead, the first and fourth authors, Luigi Micillo and Sebastian L. Kübel, should be recognized as having contributed equally to this work.
